# The Role of Plant-Associated Bacteria, Fungi, and Viruses in Drought Stress Mitigation

**DOI:** 10.3389/fmicb.2021.743512

**Published:** 2021-10-25

**Authors:** Mousami Poudel, Rodrigo Mendes, Lilian A. S. Costa, C. Guillermo Bueno, Yiming Meng, Svetlana Y. Folimonova, Karen A. Garrett, Samuel J. Martins

**Affiliations:** ^1^Department of Plant Pathology, University of Florida, Gainesville, FL, United States; ^2^Laboratory of Environmental Microbiology, Embrapa Environment, Brazilian Agricultural Research Corporation, Brasília, Brazil; ^3^Institute of Ecology and Earth Sciences, Faculty of Science and Technology, University of Tartu, Tartu, Estonia; ^4^Food Systems Institute, University of Florida, Gainesville, FL, United States

**Keywords:** microbiome, climate change, food security, plant–microbiome interaction, phytobiome, AMF, PGPR, *Arabidopsis*

## Abstract

Drought stress is an alarming constraint to plant growth, development, and productivity worldwide. However, plant-associated bacteria, fungi, and viruses can enhance stress resistance and cope with the negative impacts of drought through the induction of various mechanisms, which involve plant biochemical and physiological changes. These mechanisms include osmotic adjustment, antioxidant enzyme enhancement, modification in phytohormonal levels, biofilm production, increased water and nutrient uptake as well as increased gas exchange and water use efficiency. Production of microbial volatile organic compounds (mVOCs) and induction of stress-responsive genes by microbes also play a crucial role in the acquisition of drought tolerance. This review offers a unique exploration of the role of plant-associated microorganisms—plant growth promoting rhizobacteria and mycorrhizae, viruses, and their interactions—in the plant microbiome (or phytobiome) as a whole and their modes of action that mitigate plant drought stress.

## Introduction

Currently more than 700 million people in the world are severely affected by food insecurity according to the Food Security Information Network and Global Network Against Crises (2020), and this rate is likely to increase if the issues causing food insecurity are not addressed. Especially under climate change, drought is one of the major abiotic stresses threatening agricultural production worldwide (Lesk et al., 2016). Drought alone causes higher annual crop yield losses than all plant pathogens combined (Naylor and Coleman-Derr, 2018). In the last 35 years drought stress has reduced yields globally in staple crops such as wheat, maize, and chickpeas by as much as 21, 40, and 60%, respectively (Daryanto et al., 2016; Khan et al., 2016).

Drought affects plant–water potential and turgor, interfering with plant functions and changing plant physiological and morphological traits (Rahdari and Hoseini, 2012). Under water deficit conditions, many enzymes that participate in photosynthesis, such as ribulose-1, 5-bisphosphate carboxylase/oxygenase (Rubisco), phosphoenolpyruvate carboxylase (PEPCase), NADP-malic enzyme (NADP-ME), fructose-1,6-bisphosphatase (FBPase) and pyruvate orthophosphate dikinase (PPDK), have reduced activity (Ashraf and Harris, 2013; Hemantaranjan, 2018). Consequently, drought causes a reduction in the CO_2_ assimilation rate, malfunctioning of the primary photosynthetic reaction, and disruption of pigments, thus inhibiting photosynthetic activity in plants (Kerry et al., 2018). Other enzymes related to nutrient uptake, translocation and metabolism of nutrients are also affected by drought. One such enzyme is nitrate reductase (NR), resulting in lower uptake of nitrate from the soil (Kapoor et al., 2020). Reduction in the transpiration rate due to a water deficit also decreases plants’ absorption of nutrients and their efficiency of nutrient utilization. Thus, nutrient diffusion and mass flow of water-soluble nutrients such as nitrate, sulfate, Ca, Mg, and Si are decreased (Selvakumar et al., 2012). Additionally, drought induces free radicals that affect antioxidant defenses and reactive oxygen species (ROS), such as superoxide radicals, hydrogen peroxide, and hydroxyl radicals, resulting in oxidative stress. At high concentrations, ROS can cause plant damage, such as initiating lipid peroxidation, membrane deterioration, and degrading proteins, lipids, and nucleic acids (da Silva et al., 2013).

To cope with drought stress, compounds such as sugars, amino acids, polyols, alkaloids, and ions which contribute to photosynthesis and cell osmolarity, as well as delaying leaf senescence and enhancing root growth—are upregulated during stress (Fang and Xiong, 2015). Furthermore, synthesis of xyloglucan, expansin, pectins, lignin, and suberin was shown to increase under drought, which helps to strengthen the cell wall and maintain cell turgor (Le Gall et al., 2015). Tripathi et al. (2016) identified 163 metabolites in roots that significantly change in abundance during water stress.

Water-saving irrigation and the development of drought-tolerant cultivars through conventional plant breeding techniques and genetic engineering have been used to address drought (Eisenstein, 2013). Irrigation, however, is technical and cannot always be adopted by growers, and drought-tolerant cultivars are not always prefered by growers and consumers. Alternatively, plant-associated microbes and viruses can enhance crop productivity under climate change conditions, providing a sustainable approach to stress resistance (Xu et al., 2008; Martins et al., 2014, 2018; Nadeem et al., 2014). While many studies have elucidated how plant selection for a particular microbiome assemblage can be an important strategy for addressing biotic stressors (Mendes et al., 2011; Carríon et al., 2019), studies are just beginning to address the role of microbial communities in abiotic stress alleviation (de Vries et al., 2020). Perturbations to plant physiology caused by drought can impact the composition and metabolic activity of the plant microbiome, with consequences for both host and microbial fitness (Yang et al., 2009; Fuchslueger et al., 2016).

Drought also drives microbial responses, including biofilm formation (Khan and Bano, 2019), osmo protection (production of osmolytes) (Vurukonda et al., 2016) and morphological changes (Naylor et al., 2017; Francisco et al., 2019). Overall, drought impacts soil heterogeneity, limits nutrient mobility and access, and increases soil oxygen, often producing a substantial decrease in microbial biomass (Naylor and Coleman-Derr, 2018; Jansson and Hofmockel, 2020). Monoderm bacterial lineages such as Actinobacteria, Chloroflexi, and Firmicutes are well known to be more tolerant to desiccation due to their thicker cell walls, and their enrichment during periods of drought is driven by plant metabolism (Xu and Coleman-Derr, 2019). Drought-induced plant compounds—including sugar, amino acids, and especially G3P—are used in bacterial metabolism, and act as precursors to peptidoglycan biosynthesis in cell wall formation, enhancing the ability of monoderm taxa to improve host fitness (Xu et al., 2018; Castro et al., 2019). Semiarid environments, such as the caatinga biome, are characterized by uneven distribution of rain throughout the year (Alvares et al., 2013). Recent research has revealed the potential of bacteria associated with caatinga native plants as inoculants for plant growth promotion under drought conditions (Bonatelli et al., 2021). The adaptations of the bacteria in caatinga might involve tolerance to high temperatures, desiccation tolerance genes, pigment production for protection against UV radiation, production of thermostable enzymes, and production of intracellular osmolytes (Kavamura et al., 2013).

**FIGURE 1:**
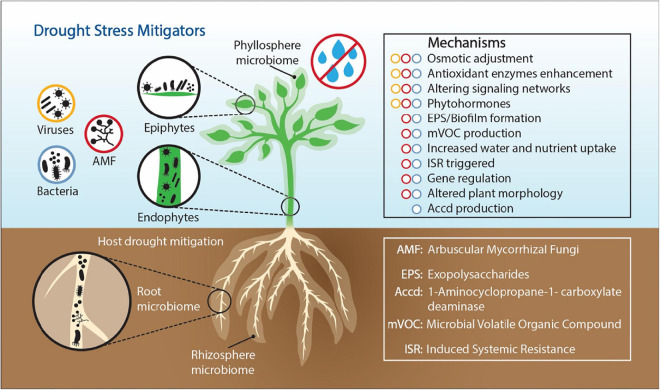
Drought stress alleviating mechanisms employed by plant-associated bacteria, fungi, and viruses. Bacteria, fungi, and viruses are members of the plant microbiome living in different plant compartments, on plant surfaces (epiphytes) and inside plant tissues (endophytes).

Exploring microbiome functions to understand how ecological processes operate during long-term drought systems provides a promising strategy to identify a subset of specific microbial taxa and functions to improve plant drought tolerance. However, whole microbiome community profiling and core microbiome assessments (composition and function), and characterizations of plant root exudates under moisture limitations, will be needed to understand relationships among environmental variables. Identifying the microbiome functions that enable plants to regulate drought stress is an important step for the development of ecological systems that will be sustainable in our future world climate. The most studied plant associated organisms are plant-growth promoting rhizobacteria (PGPR) (Ngumbi and Kloepper, 2016; Martins et al., 2018b; Ullah et al., 2019), mycorrhizal fungi (Quiroga et al., 2017; Zhang et al., 2017; Diagne et al., 2020), and viruses (Xu et al., 2008; Dastogeer et al., 2018). In this review, we focused on these three drought stress metigators (bacteria, fungi, and viruses) and explained the modes of action through which they help plants mitigate drought stress (Figure 1).

## Bacteria-Mediated Drought Tolerance

Mutualistic associations between plants and bacterial communities in the soil are very common (Naylor and Coleman-Derr, 2018) and involve rhizobacteria, bacteria present in the rhizosphere (soils in close enough proximity to the root to be influenced by root exudate release), and bacterial endophytes in the root interior (Berg et al., 2014). It has been reported that soil contains around 10^9^–10^11^ bacterial cells per gram of soil, which often not only exceeds the number of plant host cells but also the number of people existing on Earth (Berg et al., 2016). Plant-associated bacteria (PAB) can also be found aboveground in the phyllosphere, on tissues such as buds, flowers, and leaves, and within plant tissues (endophytes), as well as on pathogen survival structures (e.g., sclerotia), in which case they are termed hyperparasites (Martins et al., 2015). PAB can enhance the tolerance and resistance of plants to biotic and abiotic stresses such as pests, drought, salinity, and pH imbalances (Martins et al., 2013, 2018b; Goswami and Deka, 2020). They can be used as biocontrol agents against plant pathogens and induce production of antimicrobial compounds, such as phytohormones and siderophores, modifying the plant immune response for optimal growth (Kim et al., 2012).

Plant growth-promoting rhizobacteria, commonly known as rhizobacterial drought-tolerance enhancers (Beneduzi et al., 2012; Timmusk et al., 2013), have various mechanisms to cope with the impact of drought on plants as well as on soil. The rhizobacteria-induced drought endurance and resilience (RIDER) process involves several physiological and biochemical changes in the plant. The mechanisms by which RIDER influences plants include: (1) changes in phytohormonal activity; (2) production of aminocyclopropane-1-carboxylate deaminase deaminase (ACCd) to reduce the ethylene level in roots; (3) accumulation of osmolytes that impart drought tolerance in plants; (4) bacterial exopolysaccharide (EPS) production; (5) microbial volatiles organic compounds (mVOCs) production; (6) antioxidant defense (Porcel et al., 2014; Kumar and Verma, 2018); and (7) induction of stress responsive genes by PGPR.

### Mechanism of Action of Plant-Growth Promoting Rhizobacteria in Ameliorating Drought Stress in Plants

#### Change in the Phytohormonal Activity

Phytohormones, such as indole acetic acid (IAA), gibberellins, ethylene, abscisic acid (ABA), and cytokinin, in addition to playing a crucial role in plant growth and development, can help plants cope with abiotic stresses (Cassán et al., 2014; Porcel et al., 2014). PGPR have been reported to promote plant growth under drought conditions by manipulating plant hormones, such as cytokinins (Liu et al., 2013), ABA (Egamberdieva et al., 2017), and IAA (Jochum et al., 2019; Namwongsa et al., 2019), and decreasing ethylene production (Belimov et al., 2015).

Auxin helps ameliorate drought stress in an indirect way, by increasing root growth and/or modification of root architecture and/or root hairs (Contesto et al., 2010; Cassán et al., 2014), which favor water and nutrient uptake from the soil (Egamberdieva et al., 2017). For auxin biosynthesis, tryptophan-dependent pathways are commonly found in microbes. They produce indole-3-acetamide (IAM), indole-3-acetaldoxime (IAQx), tryptamine (TAM), and indole-3-pyruvic acid (IPA) (Su et al., 2011). Numerous reports have supported the role of IAA in triggering drought-responsible signaling pathways upon exposure to drought stress. For example, in the case of *Sorghum bicolor*, inoculation with three chromium-reducing thermo-tolerant PGPR (e.g., *Bacillus cereus* TCR17, *Providencia rettgeri* TCR21, and *Myroides odoratimimus* TCR22) improved plant growth and physiology by increasing the biosynthesis of IAA, producing plant beneficial metabolites (Bruno et al., 2020). Similarly, tomato inoculation with the PGPR *Azospirillum brasilense* caused an increase in nitric oxide content, a small diffusible gas acting as a signal molecule in IAA pathway, which caused a root morphology change in tomato (Molina-Favero et al., 2008). Hussain et al. (2014) found evidence of improved growth, biomass, and drought tolerance index in wheat seedlings inoculated with consortia of *Rhizobium leguminosarum* (LR-30), *Mesorhizobium ciceri* (CR-30 and CR-39), and *Rhizobium phaseoli* (MR-2). These modifications were correlated with enhanced auxin synthesis in the rhizobacteria-treated plants. Raheem et al. (2018) demonstrated the production of the auxins indole-3-acetic acid (IAA), indole-3-lactic acid (ILA), and indole-3-carboxylic acid (ICA) in wheat plants inoculated with *Bacillus amyloliquefaciens* S-134. These auxins triggered root growth in wheat and, thus, helped plants cope with water stress conditions. Likewise, inoculation of wheat seedlings with *Azospirillum* helped them cope with osmotic stress due to morphological modifications in coleoptile xylem architecture. These morphological modifications were associated with an upregulation of the indole-3-pyruvate decarboxylase gene, which enhanced the IAA synthesis in *Azospirillum* (Pereyra et al., 2012). A summary of phytohormonal activity associated with water stress tolerance when treated with PGPR is presented in Table 1.

**TABLE 1:** PGPR phytohormonal activity in conferring drought tolerance.

Mechanism	PGPR	Plant species	References
IAA production, increased photosynthesis	*Micrococcus luteus*	*Helianthus tuberosus*	Namwongsa et al., 2019
Increased biosynthesis of IAA and plant beneficial metabolites	*Bacillus cereus* TCR17, *Providencia rettgeri*TCR21 and *Myroides odoratimimus* TCR22	*Sorghum bicolor*	Bruno et al., 2020
Increased production of IAA, ICA and ILA	*Bacillus amyloliquefaciens* S-134	*Triticum aestivum*	Raheem et al., 2018
Increased ABA content and enhanced osmotic stress tolerance	*Phyllobacterium brassicacearum* strain STM196	*Arabidopsis*	Bresson et al., 2013
Modulation of IAA and salicylic acid	*Bacillus* sp. *Enterobacter* sp.	*Triticum aestivum, Zea mays*	Jochum et al., 2019
Modulation of phytohormones, increased metabolites	*Bacillus megaterium*	*Cicer arietinum*	Khan et al., 2019a
Modulation of phytohormones, increased metabolites	*Bacillus subtilis*	*Cicer arietinum*	Khan et al., 2019b

Under drought stress conditions, ABA modifies root architecture to enhance water and nutrient acquisition (Sah et al., 2016; Egamberdieva et al., 2017). It also sustains the hydraulic conductivity of plant roots and shoots to better exploit soil moisture and maintain cell turgor potential. Consequently, it leads to better drought tolerance through upregulation of antioxidant activities and the accumulation of compatible osmolytes, which further maintain the relative water content (RWC).

Bresson et al. (2013) reported that *Phyllobacterium brassicacearum* strain STM196, isolated from the rhizosphere of *Brassica napus*, enhanced osmotic stress tolerance in inoculated *Arabidopsis* plants by elevating ABA content, leading to decreased leaf transpiration. Guajardo et al. (2016) demonstrated that ABA treatment increased antioxidant enzyme activities, which lessened the oxidative stress-induced damage and subsequently improved drought tolerance.

#### Aminocyclopropane-1-Carboxylate Deaminase Production

Among the many PGPR products that confer plant drought tolerance, the most studied is the enzyme 1-aminocyclopropane-1-carboxylate deaminase (ACCd) (Danish and Zafar-ul-Hye, 2019; Zafar-ul-Hye et al., 2019; Danish et al., 2020). Under limited water supply, plants increase their production of stress generating ethylene (ET), which is inhibitory for plant growth (e.g., hindering seed germination and root development). Severe drought stress stimulates “1-aminocyclopropane-1-carboxylic acid” (ACC), an ET precursor that increases ethylene accumulation in plants (Gamalero and Glick, 2015). Interestingly, certain PGPR strains possess enzyme ACC deaminase (Glick et al., 2007), which can cleave the plant ethylene precursor ACC to ammonia and α-ketobutyrate, instead of converting it to ET. Therefore, ACC deaminase reduces the formation of endogenous ethylene and promotes plant growth (Chandra et al., 2019). Saleem et al. (2018) found that inoculation with ACCd producing *Enterobacter* sp. and *Bacillus* sp. increased drought tolerance in velvet bean (*Mucuna pruriens*). Niu et al. (2018) suggested that ACC deaminase and EPS-producing bacteria associated with foxtail millet (*Setaria italica*) could alleviate drought stress in plants, as indicated by improved seed germination and seedling growth. Maize inoculation with *Enterobacter cloacae* and timber waste biochar (TWBC) was found to be effective in increasing yield under drought stress, due to the potential of higher ACC deaminase synthesis, better nutrient solubilization and higher IAA production (Danish et al., 2020). Likewise, *B. amyloliquefaciens* and *Agrobacterium fabrum* promoted considerably higher yields in wheat when the plants were submitted to drought, whether inoculated individually or together. These bacteria produce ACC deaminase that catabolizes the ethylene produced in response to drought stress (Zafar-ul-Hye et al., 2019). Furthermore, the co-inoculation in *Cicer arietinum* varieties with ACC deaminase producing *Bacillus* isolate 23-B and *Pseudomonas* 6-P with *M. ciceri* improved germination, root length, shoot length, and fresh weight of chickpea seedlings under osmotic potential up to 0.4 MPa (Sharma et al., 2013).

#### Osmotic Adjustment

Plants increase the production of osmotically active molecules/ions, such as soluble sugars, proline, glycine, trehalose, betaine, organic acids, calcium, potassium, and chloride ions, as an acclimatization response to water deficit conditions (Huang et al., 2014). Proline, one of the most important osmolytes that accumulates in plants under drought stress, results in osmotic adjustment, free radical scavenging, and stabilization of subcellular structures in plant cells to overcome the detrimental effects of drought (Ngumbi and Kloepper, 2016). Some root zone bacteria release proline, which helps plants increase drought tolerance. For example, cucumber plants treated with a mixture of three PGPR strains (*Bacillus cereus* AR156, *Bacillus subtilis* SM21, and *Serratia* sp. XY21) increased leaf proline content three to four-fold, which protected the cucumber plants from over-dehydration (Wang et al., 2012). Proline content was higher under water stress when chickpea varieties were co-inoculated with *Bacillus* isolate 23-B and *M. ciceri* (Sharma et al., 2013). Similarly, increased plant drought tolerance was observed in tomato plants treated with phosphate solubilizing bacteria (PSB) *Bacillus polymyxa*, due to an increase in proline secretion (Shintu and Jayaram, 2015). Drought tolerance due to elevated proline content has been reported across several crops, such as maize (Naseem and Bano, 2014), sorghum (Grover et al., 2014), potato plants (Gururani et al., 2013), and mung bean (Sarma and Saikia, 2014), as well as *Arabidopsis* (Cohen et al., 2015). An accumulation of choline and glycine betaine (GB) due to the presence of PGPR strains *Klebsiella variicola* F2 (KJ465989), *Raoultella planticola* YL2 (KJ465991), and *Pseudomonas fluorescens* YX2 (KJ465990) also resulted in improved plant growth under drought stress (Gou et al., 2015).

Another plant drought stress tolerance mechanism provided by bacteria is the accumulation of soluble sugars due to starch hydrolysis as osmolytes. Bano et al. (2013) showed that maize seedlings inoculated with *Azospirillum lipoferum* had increased soluble sugar content and free amino acids, indicating starch hydrolysis, subsequently providing sugar for osmotic adjustment against drought. Trehalose, a xero protectant produced by bacteria under very dry conditions, works as an osmo protectant by stabilizing dehydrated enzymes and membranes (Yang et al., 2009). It also functions as a signaling molecule protecting the integrity of the cell membrane through the expression of the trehalose-6-phosphate synthase gene (Vílchez et al., 2016). A summary of the PGPR induced osmolytic activity in conferring drought tolerance is presented in Table 2.

**TABLE 2:** PGPR osmolytic activity imparting drought tolerance.

Mechanism	PGPR	Plant species	References
Accumulation of choline, glycine betaine and improved leaf relative water content (RWC) and dry matter weight (DMW)	*Klebsiella variicola* F2, *Pseudomonas fluorescens* YX2, and *Raoultella planticola* YL2	*Zea mays*	Gou et al., 2015
Accumulation of proline improved the physiological and biochemical parameters of plants	*Bacillus polymyxa*	*Lycopersicum esculentum*	Shintu and Jayaram, 2015
Expression of PEAMT gene resulted in elevated metabolic level of choline together with GB in osmotically stressed plants and improved leaf RWC and DMW	*Bacillus subtilis* GB03	*Arabidopsis*	Zhang et al., 2010
IAA induced higher proline and K-content, improved nutritional, physiological, and metabolic activities and decreased glutathione reductase (GR) and ascorbate peroxidase (APX) activity	*Bacillus thuringiensis*	*Lavandula dentata*	Armada et al., 2016
Through increased proline, abscisic acid, auxin, gibberellins and cytokinin content, improvement in plant growth	*Pseudomonas fluorescens*	*Zea mays*	Ansary et al., 2012

#### Exopolysaccharides as Drought Stress Mitigators

Exopolysaccharide, components of bacterial biofilms, provide protection against desiccation during drought stress by enhancing water retention (which may exceed 70 g water per gram of polysaccharide) and by regulating organic carbon sources diffusion (Flemming and Wingender, 2010; Chandra et al., 2021). PGPR form hydrophilic biofilm around plant roots that functions as an additional sheath to protect the roots from desiccation as well as amending the soil structure and its aggregation properties (Rolli et al., 2015). EPS released into soil as capsular and slime materials act as protective capsules around soil aggregates due to the formation of cation bridges, hydrogen bonding, Van der Waals forces, and anion adsorption mechanisms. For instance, seed treatment with *B. amyloliquefaciens* ALB629, known to produce biofilm and 5-fold more EPS compared to its absence, led to drought stress protection in common bean seedlings (Martins et al., 2018a). Additionally, maize seeds treated with EPS-producing bacterial strains *Proteus penneri*, *Pseudomonas aeruginosa*, and *Alcaligenes faecalis*, along with their respective EPS, improved soil moisture content, plant biomass, root and shoot length, and leaf area (Naseem and Bano, 2014). Yadav et al. (2018), after treating okra with rhizobia, found drought mitigation due to improved soil aggregation in the rhizosphere via sufficient EPS production. Likewise, sunflower plants treated with EPS-producing *Rhizobium* had an increase in water and nitrogen uptake, attributed to the increased root-adhering soil (RAS)/root tissue (RT) ratio and macroporosity (Vurukonda et al., 2016).

#### Role of Microbial Volatile Organic Compounds in Inducing Drought Tolerance

Microbial volatile organic compounds in bacteria are generally produced as metabolic end products of anaerobic fermentation processes and extracellular degradation of complex organic molecules (Choudhary et al., 2017). PGPR that produce mVOCs have been shown to increase seedling emergence, root branching, photosynthesis, iron uptake, plant growth, crop yield, plant disease control (Martins et al., 2019), plant parasite control (Terra et al., 2018), as well as drought tolerance. These stress-induced volatiles play a crucial role in developing priming and systemic responses in the plant producing the volatiles and in neighboring plants, making the measurement of volatiles a promising rapid non-invasive technique to assess crop drought stress and its mitigation during stress development (Niinemets, 2010; Timmusk et al., 2014).

Bacterial mVOCs mediated drought tolerance was reported by Asari et al. (2016) using *Arabidopsis* plants in which *B. amyloliquefaciens* and *B. subtilis* produced the mVOCs 3-hydroxy-2-butanone (acetoin) and 2R-, 3R-butanediol. These mVOCs increased growth in *Arabidopsis* by monopolizing gene expression involved in the maintenance of cell wall structure and influencing numerous phytohormone-signaling pathways that include ethylene, jasmonates, and salicylic acid. Timmusk et al. (2014) showed that priming treatment with *Bacillus thuringiensis* AZP2 in wheat seedlings resulted in enhanced plant biomass and five-fold higher plant survival under severe drought due to emissions of stress-related volatiles. Application of 2R, 3R-butanediol, a volatile metabolite produced by *Pseudomonas chlororaphis* O6, stimulated plant survival under drought stress by influencing stomatal aperture closure as well as inducing nitric oxide (NO) signaling in *Arabidopsis* (Cho et al., 2013). Additionally, certain bacterial mVOCs are involved in biofilm formation, which contains exopolysaccharides as major constituents, and these polysaccharides maintain soil moisture content and increase drought tolerance in plants (Naseem and Bano, 2014).

#### Antioxidant Metabolism

Water deficit conditions induce the generation of ROS in plants, including superoxide anion radicals (O_2_^–^), hydrogen peroxide (H_2_O_2_), hydroxyl radicals (OH), singlet oxygen (O_12_), and alkoxy radicals (RO). These ROS react with proteins, lipids, and deoxyribonucleic acid, causing oxidative damage and impairing the normal redox regulatory state of plant cells (Laxa et al., 2019). In such conditions, plants develop antioxidant defense systems including both enzymatic and non-enzymatic components that work to prevent ROS accumulation and protect plants from oxidative damage occurring during drought stress. Enzymatic components include catalase (CAT), superoxide dismutase (SOD), ascorbate peroxidase (APX), and glutathione reductase (GR), while non-enzymatic components contain glutathione, cysteine, and ascorbic acid (Kaushal and Wani, 2016). The presence of beneficial bacteria in soil can induce drought tolerance by modulating the antioxidant system. Experiments investigating bacterial-mediated tolerance have measured activities of antioxidant enzymes to assess the involvement of the scavenging system during drought stress. Specifically, these studies have investigated if the treatment of plants with PGPR led to a higher antioxidant enzyme activity. Moreno-Galván et al. (2020a) for instance, showed a drought tolerance in Guinea grass (*Megathyrsus maximus*) due to reduced GR activity. Likewise, the *Bacillus*–maize interactions that led to an antioxidant response to bacterial inoculation under drought stress are dependent on a decrease in APX and GR activities (Moreno-Galván et al., 2020b). Armada et al. (2016) determined that inoculation of autochthonous PGPR *B. thuringiensis* in *Lavandula dentata* and *Salvia officinalis* promoted growth and drought avoidance by depressing stomatal conductance as well as decreasing GR and APX activity. Similarly, Gururani et al. (2013) observed a 1.8-fold increase in levels of ROS-scavenging enzymes, such as CAT, APX, and SOD, in potato plants inoculated with *Bacillus pumilus* and *Bacillus firmus.* Drought tolerance driven by increased levels of CAT has also been observed in other plants, including cucumber, maize, and wheat (Sandhya et al., 2010; Wang et al., 2012). These studies provide evidence for a beneficial effect of PGPR application in conferring drought tolerance to plants by altering the antioxidant activity under drought stress conditions. A summary of antioxidant activity associated with water stress tolerance when treated with PGPR is presented in Table 3.

**TABLE 3:** PGPR antioxidant activity in imparting drought tolerance.

Mechanism	PGPR	Plant species	References
Decreased glutathione reductase (GR) activity and increased proline accumulation	*Bacillus* sp.	*Megathyrsus maximus*	Moreno-Galván et al., 2020a
Elevated secondary metabolites and peroxidase enzyme activity	*Azotobacter chroococcum Azospirillum lipoferum*	*Juglans regia*	Behrooz et al., 2019
Increased production of secondary metabolites	*Azotobacter chroococcum Azospirillum brasilense*	*Mentha pulegium*	Asghari et al., 2020
Induction of stress related enzymes, e.g., CAT, peroxidase, and ascorbate peroxidase, and lower level of H_2_O_2_ and malondialdehyde	Consortium containing *P. jessenii* R62, *P. synxantha* R81, and *A. nitroguajacolicus*YB3, *Azospirillum lipoferum* YB5	*Oryza sativa*	Gusain et al., 2015
Reduced membrane lipid peroxidation, higher enzymatic activities, higher total phenolic content	*Pseudomonas fluorescens*	*Mentha piperita*	Chiappero et al., 2019

#### Induction of Stress Responsive Genes by Plant-Growth Promoting Rhizobacteria

Stress tolerance can be enhanced by treating plants with several PGPR strains which up-regulate stress tolerance inducing genes. Vaishnav and Choudhary (2019) showed that *Pseudomonas simiae* AU promotes growth and protects soybean plants from drought stress damage, chiefly manifested as changes in the gene expression profiles of transcription factors (*DREB/EREB*), osmo protectants (*P5CS, GOLS*), and water transporters (*PIP, TIP*). *Arabidopsis* treated with *P. polymyxa* CR1 induced upregulation of two drought-responsive genes (DRGs), Response to Desiccation RD29A and RD29B (Liu et al., 2020). Additionally, Ghosh et al. (2017) found up-regulation in proline biosynthesis genes in *Arabidopsis thaliana* when inoculated with *Pseudomonas putida* GAP-P45 e.g., ornithine-Δ-aminotransferase (OAT), Δ^1^-pyrroline-5-carboxylate synthetase1 (P5CS1), Δ^1^-pyrroline-5-carboxylate reductase (P5CR), as well as proline catabolism, e.g., proline dehydrogenase1 (PDH1) and Δ^1^-pyrroline-5-carboxylate dehydrogenase (P5CDH). These changes in proline metabolic gene expression profile were positively correlated with morpho-physiological evidence of water-stress mitigation in those plants brought about by GAP-P45 inoculation under osmotic stress conditions. Likewise, the gene expression profile of pepper plants inoculated with *Bacillus licheniformis* K11 under drought stress showed higher levels of dehydrin-like protein (*Cadhn*), vacuolar ATPase (*VA*), plant small heat shock proteins (*sHSP*), and pathogenesis-related proteins (*CaPR-10*), compared to drought stress without inoculation and non-water-treated pepper (Lim and Kim, 2013). A summary of the plant genes associated with water stress response when treated with PGPR is presented in Table 4.

**TABLE 4:** Induction of stress responsive genes by PGPR in plants.

Drought responsive genes	PGPR	Plant species	References
Activation of ABA-dependent signaling genes	*Gluconacetobacter diazotrophicus* PAL5	Sugarcane plants cv. *SP70-1143*	Vargas et al., 2014
Induction of stress responsive genes such as encoding early response to dehydration (*erd15*) and encoding late embryogenesis abundant protein (*bab18*)	*Paenibacillus yonginensis* DCY84T	*Arabidopsis thaliana*	Sukweenadhi et al., 2015
Inhibition of the down regulation of ascorbate peroxidase genes, encoding RuBisCO large subunits (*rbcL*) and encoding RuBisCO small subunits (*rbcS*)	Consortium of *Bacillus cereus* AR156, *Bacillus subtilis* SM21, and *Serratia* sp. XY21)	Cucumber plants	Wang et al., 2012
Upregulation of stress related genes ascorbate peroxidase (*APX1*), (S-adenosyl-methionine synthetases (*SAMS1*), and heat shock protein (*HSP17.8*)	Priming with *Bacillus amyloliquefaciens* 5113 and *Azospirillum brasilense* NO40	Wheat leaves	Kasim et al., 2013
Up regulation of three drought stress- responsive genes, e.g., dehydration responsive element binding protein (*DREB2A*), catalase (*CAT1*) and dehydrin (*DHN*)	*Pseudomonas aeruginosa* GGRJ21	Mung plants	Sarma and Saikia, 2014
Upregulation of the transcripts of the jasmonic acid-marker genes, VSP1 and pdf-1.2, salicylic acid regulated gene, PR-1, and ethylene-response gene, HEL	*Pseudomonas chlororaphis* O6	*Arabidopsis thaliana*	Cho et al., 2013

### Screening of Drought Tolerant Microbes

Water restrictors or osmotic inducers such as polyethylene glycol (PEG) are commonly used additives in media to induce water stress. PEG is an impermeable, long-chain neutral polymer which is highly soluble in water. It produces stress in cultured cells similar to drought stress observed in cells of intact plants (Bandeppa et al., 2015; Vurukonda et al., 2016; Ali et al., 2017; Karvembu et al., 2021). These compounds reduce the osmotic potential of the culture medium, reduce water absorption and are not metabolized by plants. However, it is necessary to consider the ideal concentrations of the inducers: the ideal concentration is one which is high enough to meet the goals of the experiment and at the same time, low enough that none of the cultured organisms completely stop growing (Placide et al., 2012).

In most of the experiments, trypticase soy broth (TSB) of differing water potential prepared by adding appropriate concentrations of PEG 6000 (the number indicates the approximate molecular weight) is used to screen drought tolerant bacterial isolates (Fitriani Wangsa Putrie et al., 2013; Ali et al., 2014; Niu et al., 2018). Similarly, Martins et al. (2018) tested bacterial growth under low water activity by growing bacterial isolates in NA-broth medium altered with 2.5% (w/v) glycerol. Ansari and Ahmad (2019) determined tolerance of rhizobacteria to water stress by growing cultures supplemented with 30, 45, and 60% PEG 6000. Although the theory of classical biological control methods discusses growing bacteria in nutrient-poor media (Cook and Baker, 1983), to the best of our knowledge no studies have been conducted so far to screen drought-tolerant microbes in nutrient depleted media.

### Effect of Drought Tolerant Bacteria on Plant Health and Yield

In addition to helping plants confer drought tolerance by various mechanisms, PGPR is also reported to improve plant health and yield, which will reduce productivity losses in drought-prone areas. Raheem et al. (2018) showed that inoculation of wheat with mixed culture combinations of *B. thuringiensis* S-26, D-2, *B. amyloliquefaciens* S-134, *B. simplex* D-11, or *M. pluranimalium* S-29, *B. simplex* D-1, *B. muralis* D-5, *P. stutzeri* S-80 resulted in greater numbers of tillers and spikelets. Increased root length, shoot length, dry weight and relative water content were found in mung bean plants (*Vigna radiata*) treated with *P. aeruginosa* strain GGRJ21 (Sarma and Saikia, 2014). Likewise, Grover et al. (2014) found that treatment of *Sorghum bicolor* with *Bacillus* spp. strains KB122, KB129, KB133, and KB142 resulted in increased shoot length, root dry biomass, relative water content, sugar, chlorophyll, soil moisture content, and proline content, thereby improving sorghum seedling growth and health under drought stress conditions. Higher values of net photosynthesis (Pn), transpiration (E), and gs were observed in grapevine rootstocks K5BB plantlets inoculated with *Pseudomonas* spp. or *Delftia* spp. in comparison to uninoculated control plants (Rolli et al., 2015). Naveed et al. (2014) reported that *Burkholderia phytofirmans* inoculation of maize cultivar Mazurka improved photosynthesis (net-rate of CO_2_ assimilation under light saturation) (75%), Chl content (22%), and efficiency of PSII (10%) as compared to the control. Similarly, PGPR strains affects plant physiology by increasing RWC, Chl content, Fv/Fm and reducing leaf MDA content, thus enhancing plant growth under stress situations. Wang et al. (2016) reported that cucumber plants inoculated with a consortium of three PGPR strains called BBS (*Bacillus cereus* AR156, *Bacillus subtilis* SM21, and *Serratia* spp. XY21) reduced both MDA content as well as relative electrical conductivity and increased Chl content (Chla, band a+b increased by 25.9, 31.5, and 27.4%, respectively) in leaves over control during drought.

Studies have found that combinations of rhizobia and other PGPR improves plant health and growth under stress conditions. Co-inoculation of common bean (*Phaseolus vulgaris*) with *Rhizobium tropici*- CIAT 899, *Phaseolus polymyxa*-DSM36, and *Phaseolus polymyxa*-Loutit strains resulted in greater growth compared to inoculation with Rhizobium alone. Furthermore, co-inoculation exhibited greater nodulation (number and biomass) and nitrogen content compared to drought stressed plants inoculated with only Rhizobium (Figueiredo et al., 2008). Hussain et al. (2014) discovered that IAA produced by the consortia of *R. leguminosarum* (LR-30), *M. ciceri* (CR-30 and CR-39), and *R. phaseoli* (MR-2) improved the growth, biomass and drought tolerance index in wheat.

## Mycorrhizal Fungi-Mediated Drought Tolerance

Mycorrhizal symbiosis can be defined as the establishment of an intimate, mostly mutualistic, association between mycorrhizal fungi and plant roots (Bonfante and Genre, 2010; Smith and Read, 2010). Among the mycorrhizal fungi, arbuscular mycorrhizal fungi (AMF), belonging to the phylum Glomeromycota, are the most abundant, commonly present in agricultural soils worldwide and thought to colonize the roots of approx. 80% of land plants (Brundrett and Tedersoo, 2018; Chen et al., 2018), including most crops (Martín-Robles et al., 2018). In this symbiosis, the plant and fungus recognize each other through interacting molecular signals (Hause and Schaarschmidt, 2009; Bonfante and Genre, 2015). In particular, strigolactones exuded by plant roots stimulate branching and pre-symbiotic hypha metabolism and are considered one of the most significant components in the establishment of the association (Bonfante and Genre, 2010). This intimate association is thought to be key in the evolution of plants; ancient arbuscular-like mycorrhizal associations have been hypothesized to be responsible for the conquering of land by the first terrestrial plants (Feijen et al., 2018). This evolutionary achievement is directly related to the ability of mycorrhizal fungi to provide plants with mineral nutrients and water, along with increasing plant resistance to abiotic and biotic stresses, allowing plants to survive, develop, and diversify (Brundrett, 2002; Strullu-Derrien et al., 2018).

Central to the mutualistic nutritional role of this symbiosis is the development of arbuscules within the root cortex cells, a specialized intracellular tree-like structure, where nutrient exchange occurs (Bonfante and Genre, 2010; Smith and Read, 2010). While the AMF deliver inorganic phosphorus, nitrogen, and other benefits, the plant provides photosynthesized carbon and a secure habitat for the fungus (Brundrett, 2002; Smith and Read, 2010). One key aspect of this symbiotic nutritional pathway is that plants can reach inaccessible nutrient and water pools, as the AMF extraradical mycelia explore areas beyond the reach of fine roots and plant hairs (Smith and Read, 2010; Ferrol et al., 2019). A direct effect of this enhanced nutrient and water provision is an increase in plant fitness, i.e., growth and reproduction (Smith and Read, 2008). In agricultural terms, this translates into an overall increase of 16% grain yield (Zhang et al., 2019), in line with previous estimations of a 23, 20, and 9.5% increase in yield for tomato, wheat, and potato, respectively (Pellegrino et al., 2015; Hijri, 2016; Ziane et al., 2017). AMF can directly impact plant performance by altering the plant–water relationship, and increasing plant productivity under drought stress (Augé, 2001; Rapparini and Peñuelas, 2014). Consequently, AMF is a promising agricultural tool with the potential to decrease the use of agrochemicals, increase soil functionality, and contribute to sustainable agricultural management (Thirkell et al., 2017; Zhang et al., 2019). Despite this potential and the great amount of research devoted to understanding the diverse and complex mechanisms that regulate the AMF amelioration effects on plant water stress (Rapparini and Peñuelas, 2014; Ma et al., 2020), many key mechanistic aspects are still not understood.

### Mechanism of Action of Mycorrhiza in Conferring Drought Tolerance in Plants

**FIGURE 2:**
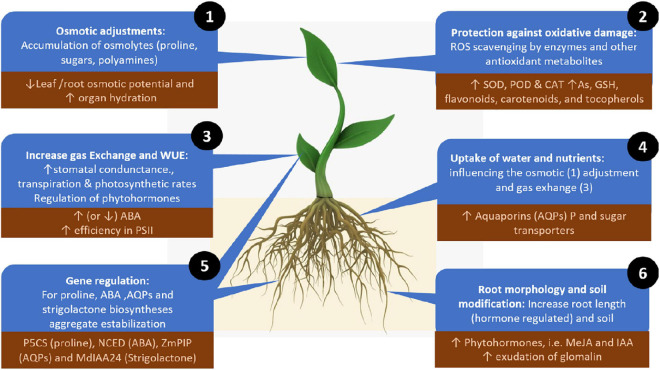
Mechanisms for alleviating drought stress employed by plant-associated arbuscular mycorrhizal fungi. Numbers depict the mechanisms in the order described in the text (order does not indicate relevance). Up or down arrows indicate increases or decreases in the regulator elements involved in each mechanism. The dark brown squared text boxes indicate specific and representative effects, molecules or genes involved in each mechanism. ROS, reactive oxygen species; AQPs, aquaporins; MeJA, methyl jasmonate; IAA, indole acetic acid; SOD, superoxide dismutase; POD, peroxidase; CAT, catalase; As, ascorbate; GSH, glutathione; ABA, abscisic acid; PSII, photosystem II.

To ameliorate the adverse effects of drought stress, AMF can directly influence plant physiology, biochemistry and genetic regulation, enhancing two main adaptive strategies: enhancing plant tolerance to drought (withstanding drought with low water potential) or avoiding drought stress by maintaining plant water status (Augé, 2001; Fang and Xiong, 2015; Ma et al., 2020). In these two strategies, AMF have several mechanisms to mediate plant responses to drought stress: (1) the uptake and transfer of water and nutrients, (2) enhanced osmotic adjustment, (3) protection against oxidative stress, (4) increased gas exchange and water use efficiency, (5) gene regulation, and (6) modification of root morphology and soil. While these roles are not completely independent, we still lack a complete understanding of the interactions among these different mechanisms (Figure 2).

#### Enhanced Osmotic Adjustment

Under drought stress, plants indirectly increase their water potential (decrease stomatal conductance), hampering the water flow from soil to the plant’s roots (Table 5). One direct mechanism is that AMF colonization can improve osmotic balance through the accumulation of inorganic osmolytes like K^+^, Ca^2+^, and Mg^2+^ (Ruiz-Lozano, 2003). However, AMF root colonization also induces biochemical changes that contribute to increased secretion of osmolytes, such as proline, polyamines (PAs), and sugars (Rapparini and Peñuelas, 2014). Each osmolyte can have different mechanisms to enhance plant drought tolerance. For example, PAs, while maintaining cellular pH and ion homeostasis, they can also scavenge ROS, stabilize the plant cell’s lipid bilayer surface and nucleic acids, and even modify stress-responsive gene expression (Talaat and Shawky, 2015; Zhang et al., 2020).

**TABLE 5:** Mechanisms of plant resistance to drought stress mediated by AMF.

Mechanism	Plant species	AMF strains	References
**Osmotic adjustments**			
Organic osmolyte accumulation	*Solanum lycopersicum*	*Rhizophagus irregularis*	Calvo-Polanco et al., 2016
	*Malus hupehensis*	*R. irregularis*	Huang et al., 2020
	*Zea mays*	*R. irregularis*	Hu et al., 2020
Ionic osmolyte accumulation	*Leymus chinensis*	*Funneliformis mosseae* and *Funneliformis geosporum*	Zhang et al., 2016
**Protection against oxidative damage**			
Higher antioxidant enzyme activities	*Setaria italica*	*Rhizophagus intraradices*	Gong et al., 2015
	*Cicer arietinum*	*R. intraradices*, *Claroideoglomus etunicatum* and *Glomus versiform*	Sohrabi et al., 2012
Higher non-enzymatic antioxidants activities	*Glycyrrhiza uralensis*	*R. irregularis*	Xie et al., 2018
Changes in polyamines concentrations	*Poncirus trifoliata*	*F. mosseae*	Zhang et al., 2020
Induced an increase in fatty acid (FA) unsaturation which alleviated oxidative damage	*Z. mays*	*R. irregularis*	Hu et al., 2020
Increased abscisic acid (ABA) that stimulating antioxidant systems	*Robinia pseudoacacia*	*R. irregularis*	Lou et al., 2021
**Increase gas exchange and water-use efficiency**			
Higher water-use efficiency (WUE) and root hydraulic conductivity	*Z. mays*	*F. mosseae*	Ren et al., 2019
Lower relative electrolyte leakage, higher stomatal conductance, total chlorophyll concentrations, net photosynthetic rate and transpiration rate	*M. hupehensis*	*R. irregularis*	Huang et al., 2020
Regulated phytohormones	*Lactuca sativa* and *S. lycopersicum*	*R. irregularis*	Ruiz-Lozano et al., 2016
**Direct uptake and transfer of water and nutrients by AM fungi**			
Grew larger external mycelia	*Cucurbita pepo*	*Glomus* sp., *Pacispora* sp., *R. intraradices*, and *Claroideoglomus claroideum*	Harris-Valle et al., 2018
Increased hyphal water absorption rate	*P. trifoliata*	*F. mosseae*	Zou et al., 2019
	*Medicago truncatula*	*R. irregularis*	Püschel et al., 2020
Increased grain number, nutrient allocation, and nutrient composition in plant	*Triticum aestivum*	*R. irregularis, F. mosseae, R. intraradices*, and *F. geosporum*	Cabral et al., 2016
**Regulation of stress-related gene expression**			
Aquaporin (AQP) genes	*P. trifoliata*	*F. mosseae*	Zou et al., 2019
ABA responsive genes	*Z. mays*	*R. irregularis*	Li et al., 2016
Proline biosynthesis genes	*P. trifoliata*	*F. mosseae* and *Paraglomus occultum*	Wu et al., 2017
FA desaturase genes	*P. trifoliata*	*F. mosseae*	Wu et al., 2019
Stomatal development gene	*S. lycopersicum*	*F. mosseae* and *R. irregularis*	
Strigolactone synthesis genes	*Malus domestica*	*R. irregularis*	Huang et al., 2021
Antioxidant genes from AMF		*Gigaspora margarita* and *R. intraradices*	Zou et al., 2021
**Root morphology and soil modification**			
Root morphological adaptation	*Abelmoschus esculentus*	*F. mosseae, R. intraradices*, and *C. etunicatum*	Ahmed et al., 2020
Increased glomalin-related soil protein	*Catalpa bungei*	*R. intraradices*	Chen et al., 2020

Particularly, proline is known to protect plant tissues against water stress by acting as an osmo solute and hydrophobic protectant. Under drought conditions, AMF induced proline accumulation has been seen in many species including cypress (*Cupressus arizonica*) (Aalipour et al., 2020), walnut (*Juglans regia*) (Behrooz et al., 2019), trifoliate orange (*Poncirus trifoliata*) (Wu et al., 2017), tobacco (*Nicotiana tabacum)* (Liu et al., 2020), and olive (*Olea europaea*) (Ouledali et al., 2018). Overall, the accumulation of proline is divergent with the degree of osmotic alterations due to AMF symbiosis and osmoregulation. AM plants accumulated higher levels of proline when exposed to drought stress compared to non-AM plants. However, some studies have also found higher leaf proline concentration in non-AMF added plants than in AMF added plants under water-stress conditions, e.g., Hazzoumi et al. (2015). This has been ascribed to less injury by water deficit, suggesting that AMF might help plants face drought by other means (Rapparini and Peñuelas, 2014). Similarly, carbohydrates have been associated with osmo-protection under drought conditions, particularly non-structural carbohydrates (e.g., sucrose, glucose and fructose) derived from AM enhanced photosynthesis (Augé, 2001). This has been observed, for instance, in trifoliate orange (Wu et al., 2017), macadamia nut (Yooyongwech et al., 2013), lettuce (Baslam and Goicoechea, 2012), and maize (Hu et al., 2020). The regulation of carbohydrates and proline does not seem to be independent: Wu et al. (2017) found that under drought stress leaf concentrations of fructose, glucose, and sucrose were higher, while in proline they were lower. This, along with evidence of stronger regulation of sucrose- and proline-metabolized enzyme activities under drought stress, indicates a potential strong effect enhancing osmotic plant adjustment to drought (Wu et al., 2017).

#### Protection Against Oxidative Stress

Drought can induce the production of excess ROS necessary the antioxidant defense system, which leads to an imbalance in cellular redox, ultimately damaging cell DNA, lipids, and proteins (Wu et al., 2014b). The antioxidant system includes enzymes (mainly SOD, POD, and CAT) and non-enzymatic molecules [e.g., ascorbate (As), glutathione (GHS), flavonoids, carotenoids, and tocopherols] (Ma et al., 2020). SOD, POD, and CAT are considered the most important members of the active oxygen scavenging enzyme system. Studies have found increased activity of SOD, POD, and CAT enzymes in plants inoculated with AMF under drought conditions (e.g., Essahibi et al., 2018; Liu et al., 2020). While the scavenging ROS effect of non-enzymatic antioxidants has been less studied, some research indicates indirect effects in reducing oxidative stress by AMF activated carotenoids, GHS and α-tocopherol (reviewed by Wu et al., 2014b; Bahadur et al., 2019).

Polyamines are implicated in growth regulation, embryogenesis, cell division, morphogenesis, and enhancing antioxidant defense systems (Altamura et al., 1991; Kumar et al., 1997; Seo et al., 2019; Vuosku et al., 2019). PAs are often composed of cadaverine (Cad), putrescine (Put), spermine (Spm), and spermidine (Spd). Inconsistency in PA accrual patterns has been observed in AM plants in response to water stress. The inoculation of water-stressed trifoliate orange with *Funneliformis mosseae* (Zhang et al., 2020) resulted in increased Put concentrations, which influenced the ROS-scavenging antioxidant mechanism (Talaat and Shawky, 2013), and decreased in Spd and Spm contents, which may be a nitrogen reserve that can be used to meet plant energy demands soon after recovering from stress (Sharma et al., 2021). In contrast to that, Hu and Chen (2020) reported significant Put content decrease in water stressed AM seedlings. The cumulative pattern of PAs may depend on the type of host plant, the AMF partners, the degree and duration of stress, and, most importantly, the developmental stages and tissues of the plants studied.

Recent studies revealed fatty acid (FA)- and ABA-related metabolites that initiated the protective responses of antioxidant systems. For example, an AMF-induced increase in the content of unsaturated FAs was found to alleviate oxidative damage in trifoliate orange (Wu et al., 2019) and maize (Hu et al., 2020), and AMF-enhanced ABA concentration was also found to stimulate antioxidant systems in black locust (Lou et al., 2021).

#### Increased Gas Exchange and Water Use Efficiency

Arbuscular mycorrhizal fungi colonization triggers plant physiological responses aboveground, including a generalized enhancement of the rates of gas exchange (stomatal conductance, transpiration, and photosynthetic rates) along with the management of plant water status (e.g., leaf water potential and relative water content) (Augé, 2001; Bárzana et al., 2012). These effects have not been shown to necessarily depend on key metabolism activities, e.g., growth or nutrition (Augé, 2001; Ruiz-Lozano, 2003). Indeed, plant water-use efficiency (WUE), the use of water in plant metabolic activities in relation to its loss in transpiration, has been shown to be highly variable in response in AM vs non-mycorrhizal plants (Augé, 2001). These have been associated with other mechanisms that are linked to water deficit, such as the management of excessive radiation via the enhanced efficiency of photosystem II by AM symbiosis (Ruíz-Sánchez et al., 2011).

Other mechanisms involved in AMF induced plant response to drought include phytohormones, where ABA plays a major role in regulating the expression of aquaporins (AQPs; membrane transporters), increasing water uptake and transport, and root hydraulic conductance (Zargar et al., 2017). Multiple studies have reported the induction of high levels of ABA by AMF root colonization under water stress (Meixner et al., 2005; Aroca et al., 2008; Ludwig-Müller, 2010; Asensio et al., 2012; Ouledali et al., 2019), but lower levels of ABA in mycorrhizal plants responding to drought have been also found (Chitarra et al., 2016; Diagne et al., 2020), indicating that the exact mechanisms are complex and remain elusive.

#### Nutrients and Water Uptake via AMF

Plant nutritional status can strongly influence the effect of drought stress in plants; indeed, the nutrient uptake by AM fungi alleviates the adverse effects of water stress on plants. Generally, mycorrhiza increased phosphorus (P) uptake under water deficiency (Augé, 2001; Püschel et al., 2021), and in return, better P nutrition enhanced by AMF may improve hydraulic conductivity and help plants to rapidly recover from water stress (Bryla and Duniway, 1997). It has been suggested that higher P content enhances photosynthetic capacity, which can lead to high stomatal conductance and transpiration (Koide, 2010). Compared to P, fewer studies have focused on the importance of AMF in nitrogen nutrition under drought stress conditions. Tobar et al. (1994) found that nitrate is made available to mycorrhizal roots directly by external mycelium under water stress. Although the influence of nitrogen supply on drought tolerance has been well documented in plants (e.g., Ding et al., 2018), the role of nitrogen contributed by AMF in hydration is still unknown.

The direct water uptake of plants via AMF under drought conditions has been questioned due to previous equivocal evidence (Smith and Read, 2010). AM fungal hyphae can penetrate soil pores inaccessible to root hairs and contribute to the redistribution of water from wetter to drier soil patches via AM fungal hyphae (Querejeta et al., 2012). This process (hydraulic lift) has been proposed to keep minimum soil moisture in surface horizons during drought, enabling both the availability of nutrients and the survival of roots and AM fungal hyphae (Smith et al., 2010). Further, the uptake and transfer of water from the soil to the plant via AMF have been experimentally demonstrated from soils with low water potential (Augé, 2001; Ruiz-Lozano, 2003). However, we know little about the relevance of this pathway, although some results are very promising. For instance, Ruth et al. (2011), using a split plant-hyphal chamber with water content sensors, demonstrated that *Rhizophagus intraradices* contributed 20% to the total water uptake, including indirectly through water effluxed from hyphae and taken up by roots. In turn, more recent greenhouse studies with trifoliate orange (*Poncirus trifoliata*) under drought vs control treatments highlights the effective role of extraradical hyphae in water absorption under drought conditions: Zou et al. (2019) observed hyphal water absorption greatly increased by 1.4 times, while Zhang F. et al. (2018) noticed elevated hyphal water absorption rate by 2.3–6.6 times.

#### Gene Regulation

As previously mentioned, the expression of drought-responsive plant genes causes the regulation of physiological responses of mycorrhizal plants to drought stress. Wu et al. (2017) reported reduced P5CS activity in AMF seedlings under drought conditions, indicating that decrease in proline accumulation in AMF seedlings is potentially associated with an AMF-modulated decrease of glutamate synthetic pathways of proline. However, inoculation with *Bradyrhizobium japonicum* in *Glycine max* and *Lactuca sativa* plants clearly indicates that induction of *P5CS* gene cannot be regarded as a AMF-induced mechanism in alleviating water stress (Porcel et al., 2004). Further studies are needed to understand these discrepancies in the regulation of *P5CS* gene in AM plants under drought. Studies have found drought treatment significantly increased the expression of *NCED* genes (Aroca et al., 2008; Merlaen et al., 2019), which encode key enzymes in ABA biosynthesis. AMF-regulated root FA desaturase genes, *PtFAD g*enes, were also observed in *P. trifoliate* under water deficiency. Similarly, AQP genes have also been described (Porcel et al., 2006; Quiroga et al., 2017). AQPs are important components of the cell transport system. It is believed that AQPs are usually associated with the symbiotic exchange process, determining the transport characteristics of plants and fungi, exhibiting diverse responses to mycorrhization (Maurel and Plassard, 2011, Jia-Dong et al., 2019). Indeed, Li et al. (2013) were the first ones to identify two functional AQP genes from the AMF *R. intraradices*, in both root cortical cells containing arbuscules and extraradical mycelia under induced osmotic stress, which highlights and confirms the fundamental and direct role of AMF in plant drought tolerance. A further step would promote mycorrhization to circumvent drought conditions in crop production. For example, more recent research has also identified a plant gene MdIAA24, which positively regulated the mycorrhization of apples by controlling the biosynthesis of strigolactone. Overexpression of this gene was associated with a general AM-induced drought-tolerant response with increased relative water content, greater osmotic adjustment, and improved gas exchange capacity among other responses (Huang et al., 2021). In turn, certain.

Antioxidant genes of AMF have shown to contribute to the mitigation of oxidative stress under drought stress (Zou et al., 2021). Together with the antioxidant systems in plants, the network of antioxidant systems in AMF are active scavengers of ROS. Similarly, a suggested mechanism by Zou et al. (2019) to maximize the plant resistance to water stress, was a complex up and down regulation of specific AQPs gene expression, although research in other species and in more natural conditions is needed to demonstrate where specific AQPs gene expression patterns are universal.

#### Modification of Root Morphology and Soil

Several studies have highlighted that AMF colonization can modulate root morphological adaptations to improve the drought tolerance of plants. These adaptations have been correlated with changes in phytohormones regulating different metabolic pathways (Wu et al., 2012, 2016; Liu et al., 2016). For instance, Wu et al. (2016) analyzed the changes in root morphology of trifoliate orange (*P. trifoliata*) seedlings treated with AMF and found a higher total root length and volume under drought stress conditions, correlated with an increase in indole-3-acetic acid, and methyl jasmonate. In the example of Wu et al. (2016), plant tolerance to drought was primarily associated with larger exploration of soil volume by roots, which allowed the plants to absorb more water and nutrients from the soil, as well as the additional AMF radical hyphae.

In addition to wider soil exploration by hyphae, AMF can induce changes in soil structure and organic matter content, which has been related to soil water retention capacity and hydraulic conductivity (Wu et al., 2014a; Bitterlich et al., 2018). Extraradical hyphae produce exudates and degradation by-products, which enhance the formation of soil aggregates, and thus modify soil aggregation, bulk density, and pore size distribution (Rillig and Mummey, 2006; Wilson et al., 2009). Some of these exudates are fungal-derived carbon compounds, e.g., glomalin, that directly contribute to soil organic matter accumulation, promoting soil organic matter storage through aggregate stabilization (Rillig et al., 2001; Wilson et al., 2009; Parihar et al., 2020). Relative to other indirect mechanisms of AMF induced drought tolerance, soil modification, despite its potential relevance, has received little attention (Querejeta, 2017). Most studies are developed in controlled conditions comparing plants with similar size, age, nutritional status, with and without AMF. Therefore, our knowledge lags about the effects of AMF community changes under more natural conditions, in which there are multiple stressors and the potential interaction of multiple microbial organisms.

#### Free-Living Symbiotic Fungi Effects on Ameliorating Drought Stress: *Trichoderma*

Besides the arbuscular mycorrhizal fungi, other fungal root endophytes, such as some strains in the genus *Trichoderma*, have parallel mutualistic roles compared to mycorrhizal fungi, enhancing plant growth and resistance to abiotic and biotic stresses (Harman and Uphoff, 2019). One main difference compared to AMF is that *Trichoderma* is relatively easy to culture, which can explain the successful earlier application of *Trichoderma* in agriculture (Field et al., 2021; Khan et al., 2021). Plant beneficial fungal strains of *Trichoderma* (hereafter *Trichoderma*) have been considered a free-living opportunistic avirulent plant symbiont (Harman et al., 2004). Among its symbiotic activities, which have historically attracted more scientific attention, are its role protecting plants against several fungal pathogens, and as a stress tolerance inducer, with important implications for sustainable crop production (Lorito et al., 2010; Harman and Uphoff, 2019). The defenseive abilities of *Trichoderma*, as an antagonist of fungal pathogens and even nematodes, have different mechanisms: the competition for space and nutrients with other fungi, production of antifungal metabolites (antibiosis), and very effective mycoparasitism. Amelioration of drought effects on plants by *Trichoderma* are thought to be a consequence of its abilities to reprogram plant gene expression through the putative activation of a limited number of general plant pathways (Shoresh et al., 2010).

#### Mechanisms of Ameliorating Drought Stress of *Trichoderma*

Plants treated with *Trichoderma* respond to drought stress by modulating physiological and biochemical parameters, which ultimately leads to the restoration of cellular homeostasis, detoxification of toxins and recovery of growth. Scudeletti et al. (2021) demonstrated that the inoculation of sugarcane plants with *Trichoderma asperellum* changed antioxidant metabolism, increasing superoxide dismutase and peroxidase enzyme activity, as well as the proline concentration and sugar portioning, and enhanced root and stalk development, thus helping plants to alleviate the negative effects of drought stress. Gusain et al. (2014) have observed enhanced drought tolerance in rice due to *Trichoderma harzianum* T35 colonization, and they indicated that *T. harzianum* promoted activity of antioxidant enzymes, SOD, CAT, and APX, thereby preventing oxidative damage to rice through quick elimination of ROS. Mastouri et al. (2012) observed that enhanced tolerance of water stress in tomato caused by *T. harzianum* T22 was due to the ability of plants to remove damaging ROS, which was accompanied by an increase in the activity of antioxidant enzymes. Mona et al. (2017) concluded that *T. harzianum* was useful in mitigating drought stress by modulation of plant secondary metabolites. The fungi improved the osmolyte proline concentration in plant tissue and increased synthesis of growth hormones, thus protecting membranes from ROS and enhancing the root system so that it can access more nutrients.

## Virus-Mediated Drought Tolerance

Viruses are submicroscopic infectious agents that are absolutely dependent on host living cells for their multiplication. In most cases, viruses have been discovered and studied in connection with the diseases they cause. Thus, they typically are regarded as pathogenic parasites, but there is increasing evidence that some viruses impart beneficial properties to their hosts. The examples range from obligate mutualistic symbiotic relationships in which viruses confer functions essential for the survival of their hosts and, therefore, the survival of the viruses themselves, to benefits that improve host performance under certain conditions (reviewed in Roossinck, 2011).

Remarkably, a number of examples illustrating beneficial effects of viruses on their hosts include cases in which virus infection promotes heat or drought tolerance in the host. For instance, virus infection of a fungal endophyte, *Curvularia protuberata*, was shown to mediate the ability of the fungus to confer heat tolerance to its host, a tropical panic grass, *Dichanthelium lanuginosum*, and, thus, survival of the host in geothermal soils in Yellowstone National Park, United States (Márquez et al., 2007). Fungal isolates cured of their symbiont, a double-stranded RNA mycovirus, *Curvularia thermal tolerance virus* (CThTV), lost the ability to confer thermal tolerance, while tolerance was restored upon virus reintroduction. Xu et al. (2008) showed that virus infection can improve drought tolerance in various plants. Infection of *Nicotiana benthamiana* plants with any of four RNA viruses—*Brome mosaic virus* (BMV), *Cucumber mosaic virus*(CMV), *Tobacco mosaic virus*, or *Tobacco rattle virus*—delayed the onset of drought symptoms when plants were kept under water-deficit conditions, compared to uninfected control plants. A similar effect was found in BMV-infected rice plants and in nine other plant species infected with CMV, including economically important crops such as tomato (*Solanum lycopersium*), watermelon (*Cucumis lanatus*), zucchini (*Cucurbita pepo*), cucumber (*Cucumis sativus*), pepper (*Capsicum annum*), and beet (*Beta vulgaris*).

### Mechanisms That Mediate the Effect of Virus Infection on Plant Drought Tolerance

#### Regulation of Osmoprotectants, Antioxidants, and Phytohormone Signaling

Transcriptome analyses of *C. protuberata* responses to CThTV infection and heat stress demonstrated that genes encoding for key enzymes in the biosynthesis and turnover of osmoprotectants such as trehalose, glycine, betaine, and taurine, as well as in the production of melanin, a pigment known to be involved in the mediation of abiotic stress in fungi, were highly induced in CThTV-caring isolates compared to the virus-free fungus in response to heat stress (Morsy et al., 2010). It is likely that such virus-induced changes in the fungus transcriptome contributed to the survival of *D. lanuginosum*, the third partner in this three-way mutualistic symbiosis found by Márquez et al. (2007) as discussed above. However, further studies will be needed to fully elucidate the virus role. Increased accumulation of several osmoprotectants, including trehalose and other sugars, as well as antioxidants, was found in metabolite profiling in CMV-infected beet and BMV-infected rice plants, which showed improved drought tolerance (Xu et al., 2008). Yet, some metabolite changes were dependent on the pathosystem. Interestingly, the overall number of metabolites affected by water withholding was less in virus-infected than in mock-inoculated plants, suggesting that the infected plants had less sensitivity to drought. A more recent study demonstrated that, in the case of CMV, drought tolerance of *A. thaliana* plants was conferred by the viral protein 2b, which interferes with plant RNA silencing pathways and also with signaling mediated by abscisic acid, the abiotic stress response hormone (Westwood et al., 2013). Water stress tolerance in *N. benthamiana* plants also increased when infected by *Yellowtail flower mild mottle virus*, manifested as a significant delay in shoot tip wilting (Dastogeer et al., 2018). The virus effect on plant gene expression and production of osmolytes and antioxidants under water-deficit conditions was similar to that of two ascomycete fungal endophytes, isolated from wild Australian *Nicotiana* species growing in a drought-prone area, which were tested along with the virus. These findings suggested that there is some similarity in the responses of fungus- and virus-infected plants to drought stress.

#### Plant Virus Evolution Under Drought Conditions Results in Increased Plant Tolerance to Drought

Changes in the environment and stress, including drought, greatly affect plant–virus interactions (Bergès et al., 2018). It seems plausible that viruses, which have an intrinsic ability to evolve rapidly, can provide new functions to hosts that enable adaptation to changing environmental conditions. Indeed, a recent study by González et al. (2021) showed that plant virus evolution under drought conditions modulated virus-plant co-adaptation, resulting in increased plant tolerance to drought. In this work, an RNA virus, *Turnip mosaic virus* (TuMV), evolved in well-watered and drought conditions by passaging in four accessions of *A. thaliana* differing in their responses to TuMV infection. Virus variants that evolved in water-deficit conditions conferred a significantly higher drought tolerance to the host plants, compared to those that evolved under the standard watering conditions. Most mutations were concentrated in the region of the virus genome that encodes VPg, a multifunctional protein involved in several aspects of virus–host interactions. The analyses of transcriptomic changes and hormonal levels in plants in response to virus infection and drought revealed that greater resistance to drought conferred by drought-evolved viruses was correlated with complex changes in gene expression and reorganization of hormone signaling. Interestingly, though, such responses were dependent on the plant accession, indicating that plant adaptation to stress involves multiple pathways. Altogether, findings from this intriguing study of experimental virus evolution under severe drought conditions provides evidence of a rapid transition from viral parasitism to mutualism taking place under stress.

## Systems Analysis to Understand the Role of the Microbiome in Drought Tolerance

A full understanding of microbiome effects on drought will support the development of more effective microbial assemblages in agriculture. It is likely that assemblages of species will be more effective in supporting drought tolerance than single species, if assemblages that support each other’s effects can be identified. Network analysis is a useful tool for identifying candidate assemblages of microbes supporting plant health, based on associations in experimental samples (Poudel et al., 2016; Garrett et al., 2018). Microbiome networks typically characterize taxa in networks in which links indicate positive (or in some cases negative) associations between taxa. Evaluating these networks can help to identify not only those microbial taxa that have positive associations with drought tolerance, but also other taxa that have positive associations with beneficial taxa, or negative associations with taxa that have negative associations with beneficial taxa (as in, “the enemy of my enemy is my friend”). Network analysis can be used to identify taxa with potentially important roles as hubs or connectors. An inherent challenge in the study of microbiome networks based on observed associations is that these associations commonly reflect factors other than biological interactions, such as shared environmental requirements, or are artifacts based on the use of relative abundance rather than absolute abundance. Network analyses can identify candidate assemblages for further empirical testing, an essential next step, to evaluate the practical economic benefits the assemblages may confer.

Machine learning offers another approach to understanding the relationships among microbiomes and drought tolerance (e.g., Poudel et al., 2019; Topçuoğlu et al., 2020). The ability of machine learning to evaluate the potential of large numbers of predictor variables is useful for integrating both the frequencies of microbial taxa and other system traits such as edaphic factors and plant phenotypic traits including transcriptomic data. An inherent limitation is the potential, when studying large numbers of taxa and transcriptomic data, that a large proportion of taxon frequencies and other potential predictors are weakly predictive of plant responses, as discussed by Efron (2020) in a recent paper about machine learning. Deep learning approaches based on neural networks are another exciting possibility as data sets expand and may include plant phenotypic traits such as plant images. Deep learning approaches have the advantage of often producing better predictions than other machine learning methods, but use of multiple machine learning tools can provide complementary perspectives.

Another methodological advance for understanding microbiomes and drought tolerance will be exploration of the use of hypergraphs to describe the microbiome and transcriptome networks for hosts and microbiomes. While typical microbiome networks are constructed based on pair-wise associations, hypergraphs include the option of links between more than two nodes. Hypergraphs have the potential to add an important new perspective on microbiome interactions.

There are also intriguing possibilities for the use of exponential random graph models (ERGMs) in the study of microbiome networks. ERGMs are often used in social network studies to evaluate network structures, such as the probability that specific types of nodes are linked (Snijders, 2002). ERGMs also have potential for evaluating the probability that particular types of taxa are associated, the network motifs describing their associations, and how these structures change from one environment to another, or from drought-tolerant to non-drought-tolerant scenarios. This type of ERGM analysis may prove useful for identifying taxa that exhibit network roles and patterns similar to other taxa known to contribute to drought tolerance. These similar patterns may be indicative of the importance of these taxa: potentially they have similar beneficial roles, or are competitors of beneficial species, etc.

Combining these tools in ensemble models, and evaluating their results side by side, will provide important perspectives on microbial assemblages to support drought tolerance. The hypotheses about key taxon combinations generated by systems analysis will support the next steps for empirical testing.

## Conclusion and Future Prospects

Plant microbiomes have the potential to confer tolerance to abiotic and biotic stresses, including drought. The application of single microbes, consortia of several microbial isolates or the manipulation of the crop microbiome represent promising strategies for addressing many of the challenges of drought to agricultural productivity. Multiple traits of microbes should be considered to identify more efficient inoculants for future use in agriculture. As previously proposed for biotic stress tolerance (Mendes et al., 2013), we suggest the assembly of a “minimal microbiome,” comprised by a minimal set of microbial traits needed to provide a specific ecosystem service, in this case tolerance to drought stress. One way to do this is through microbiome engineering (Kaushal and Wani, 2016). To make progress on this, it is essential to understand the chemical signaling and molecular mechanisms through which PGPR and microbiomes affect plants and interact with them during stress. In addition, drought-tolerant bacteria and microbiomes associated with crop species that are naturally adapted to drought should be given more emphasis. Some of these microbes produce non-reproductive structures, such as endospore in the phylum Firmicutes, which can survive under drought conditions for a long period of time, making them good candidates for formulation to be applied in dry areas.

Viruses, though regarded as pathogenic parasites, interestingly can confer drought tolerance to host plants. However, a very limited number of known mechanisms for viruses to provide drought tolerance are known compared to PGPR and AMF. It is also important to unravel the interactions among these organisms as they aid plants exposed to drought stress, and to understand the chemical signaling and molecular mechanisms through which they interact with plants during stress.

## Author Contributions

MP wrote the first draft and revised the manuscript into its final format. SM presented the original idea, worked with MP on the first draft, worked on Figure 1 with MP, and edited the final version. RM and LC mainly contributed to the discussion of the role of the whole microbial community in mitigating drought stress, CB and YM contributed to the discussion of arbuscular mycorrhizae and *Tricoderma*, including Figure 2 and Table 5. KG contributed the discussion of systems analysis to understand microbial roles in drought tolerance. SF contributed to the discussion of virus-mediated drought tolerance. All authors contributed to the writing and edited the final versions and approved the submitted version.

## Conflict of Interest

RM and LC are employed by the Brazilian Agricultural Research Corporation. The remaining authors declare that the research was conducted in the absence of any commercial or financial relationships that could be construed as a potential conflict of interest.

## Publisher’s Note

All claims expressed in this article are solely those of the authors and do not necessarily represent those of their affiliated organizations, or those of the publisher, the editors and the reviewers. Any product that may be evaluated in this article, or claim that may be made by its manufacturer, is not guaranteed or endorsed by the publisher.
